# Performance of conventional pigs and Göttingen miniature pigs in a spatial holeboard task: effects of the putative muscarinic cognition impairer Biperiden

**DOI:** 10.1186/1744-9081-9-4

**Published:** 2013-01-10

**Authors:** Elise Gieling, Welmoed Wehkamp, Remco Willigenburg, Rebecca E Nordquist, Niels-Christian Ganderup, Franz Josef van der Staay

**Affiliations:** 1 Emotion & Cognition Group, Department of Farm Animal Health, Faculty of Veterinary Medicine, University Utrecht, P.O. Box 80151, Utrecht, The Netherlands; 2 Rudolf Magnus Institute of Neuroscience, Utrecht University, Universiteitsweg 100, Utrecht, 3584 CG, The Netherlands; 3 HAS Den Bosch, University of Applied Sciences, Postbus 90108, ‘s-Hertogenbosch, The Netherlands; 4 Ellegaard Göttingen Minipigs A/S, Soroe Landevej 302, Dalmose, DK-4261, Denmark

**Keywords:** Working memory, Reference memory, Animal model, Holeboard task, Spatial learning task, Biperiden

## Abstract

**Background:**

The pig is emerging as a model species that bridges the gap between rodents and humans in research. In particular, the miniature pig (referred to hereafter as the minipig) is increasingly being used as non-rodent species in pharmacological and toxicological studies. However, there is as yet a lack of validated behavioral tests for pigs, although there is evidence that the spatial holeboard task can be used to assess the working and reference memory of pigs. In the present study, we compared the learning performance of commercial pigs and Göttingen minipigs in a holeboard task.

**Methods:**

Biperiden, a muscarinic M1 receptor blocker, is used to induce impairments in cognitive function in animal research. The two groups of pigs were treated orally with increasing doses of biperiden (0.05 – 20 mg.kg^-1^) after they had reached asymptotic performance in the holeboard task.

**Results:**

Both the conventional pigs and the Göttingen minipigs learned the holeboard task, reaching nearly errorless asymptotic working and reference memory performance within approximately 100 acquisition trials. Biperiden treatment affected reference, but not working, memory, increasing trial duration and the latency to first hole visit at doses ≥ 5 mg.kg^-1^.

**Conclusion:**

Both pig breeds learned the holeboard task and had a comparable performance. Biperiden had only a minor effect on holeboard performance overall, and mainly on reference memory performance. The effectiveness needs to be evaluated further before definitive conclusions can be drawn about the ability of this potential cognition impairer in pigs.

## Introduction

Although most preclinical studies in neurosciences are performed using rodents, in particular mice, the pig is increasingly being used as a model species because it can bridge the anatomical/physiological gap between rodents and humans [1-3]. In particular, the miniature pig (referred to hereafter as the minipig) is gaining popularity as laboratory animal in pharmacological and toxicological studies [4]; however, pigs have not been used extensively in behavioral studies, mainly because there are few validated behavioral tests for pigs. Recent systematic reviews have found pigs able to acquire a broad range of learning and memory tasks [5-7], such as the holeboard task [8,9].

The holeboard is a food-rewarded maze, where bait can be found in different places, and the animal is free to visit these places in whatever order it chooses. Once an animal has visited a place and consumed the food, that place is not rebaited during a trial and thus return visits to the same location are not reinforced (reviewed in [10]). If food can only be found in a subset of potential sites, then two memory components can be distinguished and measured simultaneously: spatial working memory (WM) and reference memory (RM) [11]. WM holds information that is relevant only within a specific trial, such as a list of locations that have recently been visited/explored. An animal thus must process the temporal context associated with an event – “what happened, and when did it happen” – and keep in mind which locations have already been visited, in order to efficiently deal with the spatial WM component of a task [12]. The information is transiently held in memory until the specific trial has been completed and is of no value for performing the next trial. RM holds information about the solution of the spatial holeboard discrimination task, such as the localization of the food and the actions necessary to get the bait [13], for example, lifting up a ball covering each food bowl with the snout [9]. RM thus stores the general rules of a task. It retains relevance across many trials and is thus trial independent, but learning task specific. Little is known about the ability of Göttingen minipigs to learn the holeboard task. Manton [14] tested minipigs in a holeboard at the ages of approximately 59, 88, and 93 days, on each occasion over 2 consecutive days with 3 training trials per day, and found the animals to have a very poor WM and RM performance, with RM performance hardly exceeding chance level.

Central cholinergic neurotransmission appears to be involved in spatial learning and memory processes [15]. Of the five known muscarinic receptor subtypes, M1, M2, M4, and M5 are found in the human brain. M1, M2, and M4 receptors are abundant in the hippocampus and cortex, areas that that involved in learning and memory. Blockade of these receptors thus is expected to induce cognitive deficits [16]. The non-selective muscarinic receptor antagonist scopolamine is used to induce cognitive impairments in animal models of disorders characterized by cognitive dysfunctions [17], such as Alzheimer’s disease. Bouger and van der Staay [18] showed that administration of scopolamine (or the non-competitive NMDA antagonist MK-801) in well-trained rats transiently, but consistently, impaired WM and RM in a conefield, a variant of the holeboard. Recently, it has been suggested that biperiden, a muscarinic M1 receptor antagonist [16], may be better suited to induce cognitive deficits in animal experiments [19]. Biperiden (manufactured as Akineton by BASF/Knoll Pharma, New Jersey, USA) is generally used as an antiparkinsonian agent in humans. In humans, the drug has a poor availability of only 13% after oral administration; its bioavailability after systemic administration is 33% (+/- 5%). The t_max_ of biperiden is 0.5–1.5 h and the t_½_ is 21 h (+/- 3.1 h). [20] Biperiden appears to be tolerated in high dosages before overt and lethal toxicity occurs: an oral LD_50_ of 750 mg.kg^-1^ has been reported for rats and 340 mg.kg^-1^ for dogs [21]. Common observable side effects and neuropsychiatric signs include dry mouth, drowsiness, agitation, anxiety, hyperactivity, ataxia, and loss of memory [22].

In line with the expectation that M1 receptor blockade would impair cognition, biperiden-induced cognitive deficits have been observed in different species, including humans [23]. In humans, motor learning as well as visuospatial processes were impaired after oral administration of 2 mg biperiden [23]. Silver et al. [24] observed that biperiden (2 mg twice daily) given to schizophrenic patients impaired performance on the Benton Visual Retention Test and the visual subscale of the Wechsler Memory Scale. Similarly, Liang et al. [25] showed that patients with schizophrenia treated twice daily with 2 mg biperiden had impaired cognitive functions, tested using the Mini Mental State Examination (MMSE). In rats, biperiden has been found to impair responding at 10 mg.kg^-1^ and to impair short-term memory in a series of operant learning and memory tasks [19], but it did not affect food motivation or attention. In another study, biperiden (4, 8, or 16 mg.kg^-1^, injected intraperitoneally) delayed consolidation in a passive avoidance task [26].

As scopolamine is a non-selective muscarinic receptor antagonist, it is difficult to establish whether its behavioral effects are mediated centrally (cognitive) or peripherally (side effect). Biperiden might be a better choice because it is more selective. Moreover, scopolamine is usually administered intraperitoneally or intramuscularly [17], but in pigs these routes of administration might be unduly stressful, which could interfere with learning [27]. In contrast, biperiden is administered orally. Oral administration in food is the least stressful and preferred way to administer drugs to pigs provided that the drug-containing food does not induce food aversion and is palatable [28]. As we would like to know whether Göttingen minipigs are suitable for studying cognitive function, we tested whether 1) conventional pigs and age-matched Göttingen minipigs learn the holeboard task at a similar speed and to a similar asymptotic performance level, and 2) whether orally administered biperiden transiently impairs spatial memory in pigs. We expected that the Göttingen minipigs would be able to learn the holeboard task as good as conventional pigs do and that biperiden would impair cognition in both pig lines, as was found earlier with rats [19]. We expect biperiden to be an interesting alternative for scopolamine for inducing cognitive impairment.

## Material and methods

### Ethical approval

The experiments were reviewed and approved by the local ethics committee (DEC, **d**ier**e**xperimenten**c**ommissie) and were conducted in accordance with the recommendations of the EU directive 86/609/EEC. All efforts were made to minimize the number of animals used and to avoid suffering.

### Animals

Eight female Göttingen miniature pigs (supplier: Ellegaard Göttingen Minipigs A/S, Dalmose, Denmark) and 8 female piglets [Duroc X (Fin X York)] born at the pig-breeding farm of Utrecht University were used. Healthy piglets from different litters were selected after weaning and were moved in a covered trolley to our experimental facility when they were 4–6 weeks old. The minipigs were transported from Denmark to the Netherlands in a climate-controlled minivan.

### Housing

The pigs were housed together per breed in two adjacent identical pens of 20 m^2^, situated in a large, naturally ventilated and lit stable. The pens had a concrete floor, covered with straw bedding; drinking water was provided *ad libitum*. A covered piglet nest, a play ball, and chewing sticks were provided per pen. In addition, a heating mat (25°C) covered with straw was installed in the nest of the Göttingen minipigs. All pigs were fed twice a day, once in the early morning (1/3 of their daily feed ration in the morning, about 1 hour before testing started) and once in the late afternoon (2/3 of their daily feed ration after completion of daily holeboard training). The Göttingen minipigs were fed on a diet according to the recommendations of the breeder. All pigs were weighed at least once a week.

### Testing room

The testing equipment was located in the room next to the pens. The testing room consisted of a corridor, a waiting area, and the holeboard apparatus. All pigs from one group were walked down the corridor and entered the straw- and toy-enriched waiting area (11.5 m^2^, *ad libitum* access to drinking water) of the testing apparatus. Then the experimenter let one pig into the testing apparatus. After testing, the pig returned to the waiting area.

### Drug

Akineton tablets (containing 2 mg Biperiden; producer: Abbott Laboratories) were crushed and mixed with conventional pig food, honey (to make it more palatable), and some water into a ball.

### Apparatus

The holeboard consisted of a square arena measuring 530 by 530 cm, with a 4×4 matrix of food bowls (for a schematic overview of the apparatus see [9]). The blue synthetic floor was slatted and the gray synthetic walls (height: 80 cm) had a steel bar on top (at a height of 100 cm). The arena had four entries (one on each side) through guillotine doors that were operated from the outside by the experimenter using pulley strings. By walking down a small corridor (width: 40 cm) surrounding the entire arena, the animals found the opened door and entered the holeboard on their own initiative. The apparatus (arena and corridor) was elevated above the floor. The testing room provided adequate extra-maze cues.

To prevent the pigs from locating rewards based on smell, all food bowls had false bottoms under which fresh rewards (M&M milk chocolates®) were placed daily. To prevent the animals from locating the rewards visually, each bowl was covered with a synthetic red ball (Jolly Ball Dog Toy, diameter: 24 cm, weight: 400 g). The pigs could get at the reward by lifting the ball with their snout; the ball rolled back on the bowl as soon as the pig withdrew its head. The apparatus was cleaned with water before the next trial, and the entire apparatus was rinsed with water daily after use.

### Habituation

When the pigs were approximately 10 weeks old, they were gradually exposed to their handlers, the testing room, and the apparatus, as described by Gieling et al. [9]. During the first week of habituation, the pigs were allowed to get used to the experimenters and the waiting area, and from the second week they were habituated to the holeboard. The pigs had four daily sessions of 20 minutes per pen. The animals were first habituated to the holeboard in groups of 8 pigs, then in groups of 4 pigs, and lastly in groups of 2 pigs. During the habituation period, all food bowls contained M&Ms. Individual pigs were habituated to the holeboard during four trials/day on 10 successive working days until the pig had found all rewards or 10 minutes had passed, whichever event occurred first. The next working day, training was started.

### Acquisition

When the pigs were about 13 weeks old, formal training in the holeboard started. Each animal was assigned its own configuration of four rewarded holes. Four different configurations were used (determined using the rule outlined in Figure 4K in [10]: a basic configuration (Figure 4E in [10]) and its three rotations (90, 180, and 270 degrees). In this way, all sixteen holes were rewarded equally often. The entrance door was randomly chosen for each trial. Each pig received 2 acquisition sessions of 2 trials each day (1 session in the morning and 1 session in the afternoon) for the first 13 working days (i.e. 26 sessions). Then, from working day 14 to 40, each daily session consisted of 2 massed trials. The acquisition phase consisted of 104 trials. Testing was never performed during weekends.

### Data collection

Data collection was automated. Each food bowl was equipped with a hidden sensor. If the ball (which was fitted with a magnet) on top of the bowl was lifted, a signal was broken and sent to an interface (LabJack) and stored on a Personal Computer (OS: Windows XP), using the custom made software ‘Experiment control for University Utrecht’ (Blinq Systems, Delft, The Netherlands). A trial ended automatically after an animal had found all rewards or 10 minutes had elapsed.

### Drug treatment

After they reached asymptotic performance, all pigs were treated with increasing doses of biperiden (0.05 mg.kg^-1^, 0.15, 1.5, 5, 15 and 20 mg.kg^-1^; the lowest dose corresponds to the therapeutic dose in humans) administered orally 1.5 hours before holeboard testing started. As in the training trials, testing consisted of 2 trials in close succession. At 8:30, the first pig was given its ball of food containing biperiden in a food bowl (to prevent spoiling), followed at 15-minute intervals by the other pigs in random order. This was the order in which the pigs were tested. Pigs were given the rest of their morning ration of feed 30 minutes after administration of the two lowest doses of biperiden, to ensure that their performance was not altered by hunger. This was not necessary for the higher doses, because the drug-feed mixture contained the pigs’ normal feed ration. Between each drug-testing session, there was a wash-out period of at least 2 days, based on the t_½_ of 18.4–24.3 hours of biperiden in humans and rodents. The pigs continued to train during the wash-out periods. Biperiden was tested in sessions 22 (0.05 mg.kg^-1^), 32 (0.15 mg.kg^-1^), 36 (1.5 mg.kg^-1^), 39 (5 mg.kg^-1^), 41 (15 mg.kg^-1^), and 44 (20 mg.kg^-1^).

### Drug-induced side effects

The behavioral side effects of biperiden [29] were registered by two researchers while the pigs were in the waiting area. Behavioral sedation was scored when an animal lay down 1 minute or longer. Dry mouth (shown by yawning-like behavior) and dry cough were scored if these behaviors were observed more than twice.

### Statistical analysis

WM and RM are expressed as ratios [10]. WM was defined as the number of rewarded visits divided by the number of visits to the baited set of holes. This ratio reflects the ability of the animals to avoid re-visits to baited holes during a trial. RM was defined as the number of visits to the baited set of holes divided by the number of visits to all holes. This ratio reflects the ability of animals to discriminate between baited and unbaited holes.

To analyze differences in the speed of holeboard task acquisition by the two groups of pigs, the means of blocks of 4 trials (1 testing day) were calculated. Changes in WM, RM, trial duration, and latency to first rewarded hole visit in the course of training were assessed by an analysis of variance (ANOVA) with the between subjects factor Pig breed (conventional pigs vs. Göttingen minipigs) and the within subjects (repeated measures) factor Blocks of Trials (SAS GLM procedure, SAS Institute, Cary, NC, USA). To analyze the effect of acute challenge with biperiden, means were calculated for both drug and drug-free sessions. Both session means consisted of two successive trials. First we tested whether the drug-free sessions differed from each other (repeated measures ANOVA) to decide whether an overall drug-free session mean could be used or whether separate sessions should be analyzed. To assess whether biperiden affects holeboard behavior, and whether this effect is different for the two pig breeds, we performed a Pig breed (Conventional pigs vs. Göttingen minipigs) by Doses (0.05, 0.15, 1.5, 5, and 15 mg.kg^-1^ Biperiden) by Sessions (Control session preceding Biperiden treatment vs. Biperiden session) ANOVA with repeated measures on the second and third factors. Unfortunately, one of the conventional pigs did not eat the 15 mg.kg^-1^ dose of biperiden, so the data for this pig were omitted from the repeated measures analysis. Because the conventional pigs did not eat the entire of 20 mg.kg^-1^ dose of biperiden, only the doses up to 15 mg.kg^-1^ were considered for comparisons between pig breeds. In addition, we analyzed the effects of biperiden *in* the Göttingen minipigs by a Doses by Sessions repeated measures ANOVA for all doses tested (i.e. including the 20 mg.kg^-1^ biperiden dose). An alpha of < 0.05 was considered significant.

## Results

### Acquisition of the holeboard task

#### Working memory

**Figure 1:**
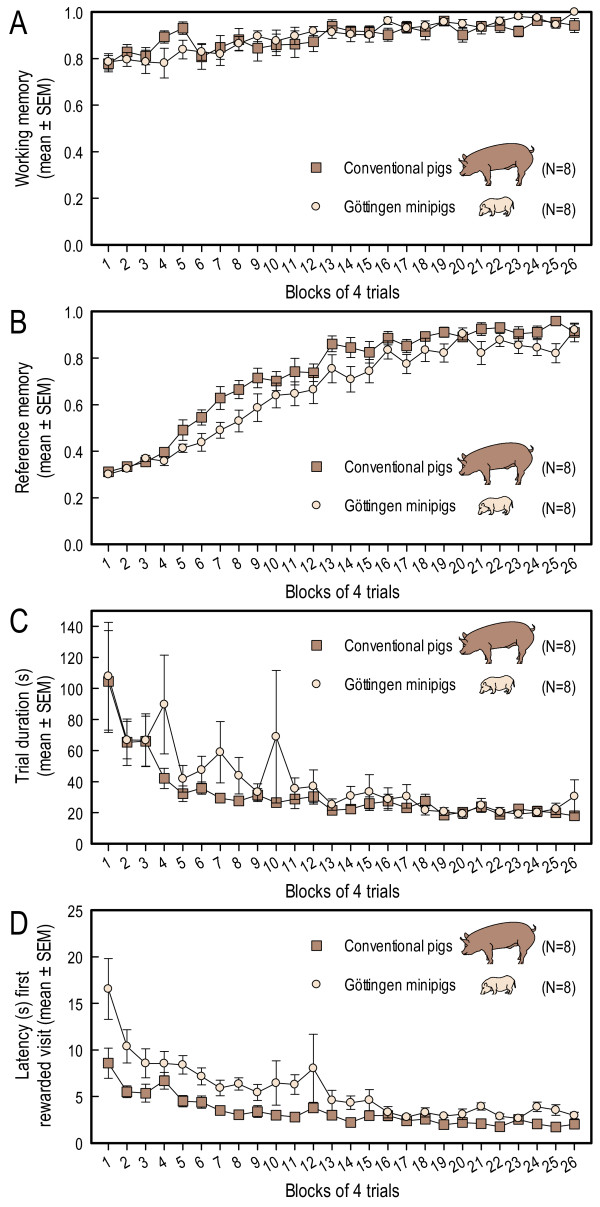
**Learning of a spatial holeboard discrimination task by conventional pigs and Göttingen minipigs.** The working memory performance (panel **A**) and the reference memory performance (panel **B**), the trial duration (panel **C**) and the latency to first rewarded hole visit (panel **D**) are depicted as means and standard errors of the mean (SEM) of 26 successive blocks of 4 trials each.

WM performance (see Figure 1A) was similar in the two groups of pigs (F_1,14_ = 0.02, p = 0.8939) and improved with training (Blocks of trials: F_25,350_ = 7.41, p < 0.0001) similarly in the two groups of pigs (Pig breed X Blocks of trials interaction: F_25,350_ = 1.10, p = 0.3607).

#### Reference memory

The Göttingen minipigs tended to have a poorer RM (see Figure 1B) than the conventional pigs (F_1,14_ = 4.02, p = 0.0646). RM performance improved across blocks of trials (Blocks of trials: F_25,350_ = 104.43, p < 0.0001), and while learning appeared to be slightly delayed in the Göttingen minipigs (Pig breed X Blocks of trials interaction: F_25,350_ = 1.44, p = 0.0811), both groups of pigs ultimately had a similar level of performance.

#### Trial duration

The two groups of pigs completed the trials at a similar speed across all trial blocks (F_1,14_ = 1.31, p = 0.2708), and trials became shorter in the course of learning (Blocks of trials: F_25,350_ = 6.51, p < .0001) in both groups of pigs (Pig breed X Blocks of trials interaction: F_25,350_ = 0.66, p = 0.8910). This is shown in Figure 1C.

#### Latency to first rewarded hole visit

As is shown in Figure 1D, the conventional pigs gained their first reward faster than the Göttingen minipigs (F_1,14_ = 12.39, p < 0.0034), but pigs in both groups became quicker in finding the food reward in the course of training (Blocks of trials: F_25,350_ = 12.61, p < 0.0001) (Pig breed X Blocks of trials interaction: F_25,350_ = 1.67, p = 0.0252). Whereas the Göttingen minipigs needed more time to find the first reward than the conventional pigs during the first block of four trials (t_14_ = -2.19, p = 0.0462), the performance of both lines was similar during the last block of four trials (t_14_ = -1.64, p = 0.1241).

### Effects of biperiden

#### Side effects

**Figure 2:**
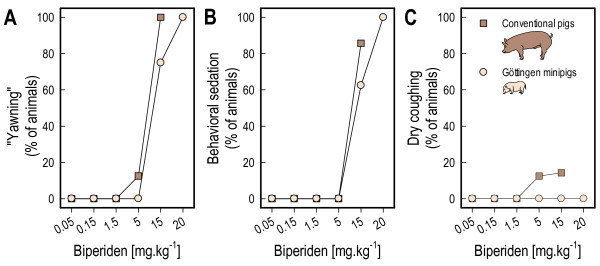
**Side effects of biperiden at different doses.** Panel **A**: percentage of animals with signs of dry mouth (“yawning”). Panel **B**: percentage of animals showing signs of behavioural sedation. Panel **B**: percentage of animals with dry cough. As the conventional pigs did not eat all of the 20 mg/kg dose, the side effects of this dose are excluded for this group (Note: legend in panel **C** also applies to panels **A** and **B**).

Most pigs showed signs of a dry mouth (xerostomia) and mild behavioral sedation (lying down for longer than normal in the waiting area) with the higher doses of biperiden (≥ 15 mg.kg^-1^) (Figure 2).

#### Control (drug-free) sessions

Performance during the drug-free sessions that preceded test days did not change, with the exception of RM (F_5,70_ =2.92, p = 0.0190). Contrast variables showed that for RM only sessions 1 and 2 (F_1,14_ = 5.11, p = 0.0403) and 3 and 4 (F_1,14_ = 9.29, p = 0.0087) differed from each other. On the basis of these results, we decided to compare a treatment session with its own preceding drug-free session and not with the average of all drug free sessions preceding a treatment session.

#### Working memory

**Figure 3:**
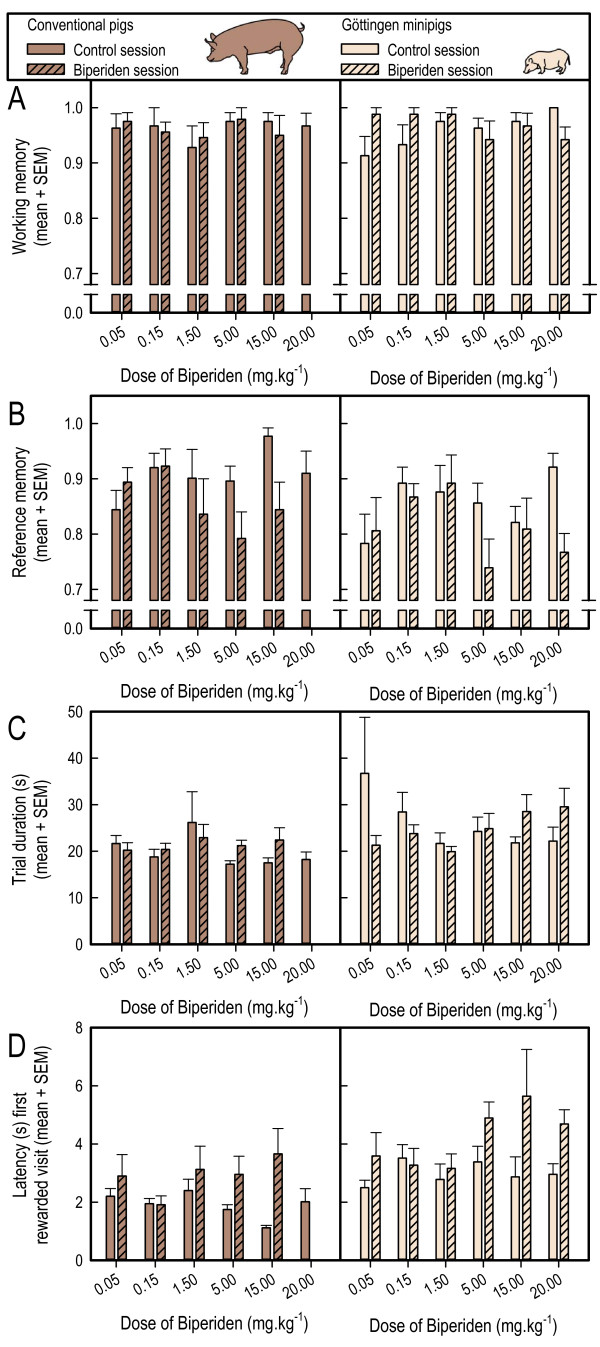
**Effects of oral administration of increasing doses of biperiden on working memory (A) and reference memory performance (B), trial duration (C), and latency to first rewarded hole visit (D) of conventional pigs and Göttingen minipigs.** The means + SEM of the drug-free day preceding treatment and of the day of biperiden treatment are depicted. None of the conventional pigs consumed the entire 20 mg.kg^-1^ dose. Consequently, these data were not analyzed, and only the data of the Göttingen minipigs are shown for the highest dose of Biperiden.

The WM performance (see Figure 3A) of the two groups of pigs, averaged over all doses up to 15 mg.kg^-1^ and sessions, was not significantly different (F_1,13_ = 0.00, p = 0.9595), and biperiden did not affect WM (Dose: F_4,52_ = 0.03, p = 0.9979; Pig breed X Dose interaction: F_4,52_ = 0.89, p = 0.4742; Session: F_1,13_ = 0.62, p = 0.4446.; Pig breed X Sessions interaction: F_1,13_ = 0.30, p = 0.5952; Dose X Sessions interaction: F_4,52_ = 1.55, p = 0.2012; Pig breed X Dose X Sessions interaction: F_4,52_ = 0.52, p = 0.7189). However, analysis of the data for all biperiden doses tested (0.05–20 mg.kg^-1^) revealed that biperiden affected WM in the minipigs (Doses X Sessions interaction: F_5,35_ = 3.25, p = 0.0163; Doses: F_5,35_ = 0.46, p = 0.8020; Sessions: F_1,7_ = 0.36, p = 0.5666).

#### Reference memory

The RM performance (see Figure 3B) of the two groups of pigs was similar, averaged over all doses and sessions (F_1,13_ = 2.00, p = 0.1807), and biperiden treatment affected RM performance (Dose: F_4,52_ = 3.73, p = 0.0096) similarly in the two groups (Pig breed X Dose interaction: F_4,52_ = 0.69, p = 0.5995; Session: F_1,13_ = 4.66, p = 0.0501; Pig breed X Sessions interaction: F_1,13_ = 0.44, p = 0.5172; Dose X Sessions interaction: F_4,52_ = 2.53, p = 0.0511; Pig breed X Dose X Sessions interaction: F_4,52_ = 1.17, p = 0.3339). Note that the Sessions effect, i.e. the overall performance in the control sessions vs. the overall performance in the drug treatment sessions, had an associated probability close to 0.05. This suggests that, on average, the pigs performed worse in the biperiden sessions. The marginal Dose X Sessions interaction suggests that the impairment induced by biperiden was dose dependent. Analyses revealed a marginal dose-related effect of biperiden in the minipigs (Doses X Sessions interaction: F_5,35_ = 2.21, p = 0.0748, Doses: F_3,35_ = 3.64, p = 0.0093; Sessions: F_1,7_ = 4.21, p = 0.0792), with higher doses (except 15 mg.kg^-1^) appearing to decrease RM performance (see Table 1).

**Table 1:** Biperiden effects per pig line and dose of Biperiden

**Biperiden (mg.kg**^**-1**^**body mass, p.o.)**	**Conventional pigs**		**Göttingen minipigs**		**Conventional pigs**		**Göttingen minipigs**	
	**Working memory**				**Reference memory**			
**0.05**	t_8_ = 0.36	p = 0.7318	t_8_ = 2.05	*p = 0.0796*	t_8_ = 1.18	p = 0.2765	t_8_ = 0.34	p = 0.7425
**0.15**	t_8_ = -0.24	p = 0.8157	t_8_ = 1.63	p = 0.1477	t_8_ = 0.09	p = 0.9332	t_8_ = -0.66	p = 0.5277
**1.5**	t_8_ = 0.39	p = 0.7055	t_8_ = 0.55	p = 0.5983	t_8_ = -1.43	p = 0.1966	t_8_ = 0.27	p = 0.7986
**5**	t_8_ = 0.14	p = 0.8916	t_8_ = -0.62	p = 0.5577	t_8_ = -2.96	***p = 0.0212***	t_8_ = -4.75	***p = 0.0021***
**15**	t_7_ = -0.46	p = 0.6585	t_8_ = -0.41	p = 0.6975	t_7_ = -2.41	*p = 0.0528*	t_8_ = -0.22	p = 0.8316
**20**	n.t.		t_8_ = -2.50	***p = 0.0412***	n.t.		t_8_ = -3.55	***p = 0.0094***
	**Trial duration**				**Latency to first rewarded hole visit**			
**0.05**	t_8_ = -0.81	p = 0.4427	t_8_ = -1.31	p = 0.2325	t_8_ = -1.00	p = 0.3519	t_8_ = -1.25	p = 0.2522
**0.15**	t_8_ = 0.89	p = 0.4032	t_8_ = -1.45	p = 0.1895	t_8_ = 0.08	p = 0.9375	t_8_ = 0.29	p = 0.7797
**1.5**	t_8_ = -0.49	p = 0.6358	t_8_ = -0.78	p = 0.4612	t_8_ = -1.18	p = 0.2749	t_8_ = -0.74	p = 0.4831
**5**	t_8_ = 4.27	***p = 0.0037***	t_8_ = 0.21	p = 0.8401	t_8_ = -1.63	p = 0.1476	t_8_ = -1.98	*p = 0.0876*
**15**	t_7_ = 1.55	p = 0.1725	t_8_ = 2.05	*p = 0.0792*	t_7_ = -3.00	***p = 0.0241***	t_8_ = -1.57	p = 0.1614
**20**	n.t.		t_8_ = 1.98	*p = 0.088*7	n.t.		t_8_ = -5.11	***p = 0.0014***

One-sample t-statistics and the associated p-values of the difference scores between the control sessions preceding treatment and the corresponding treatment sessions are tabulated. Abbreviation: n.t. not tested, because none of the conventional pigs consumed the entire dose of 20 mg.kg^-1^. p-values < 0.05 are printed bold and italicized, p-values between 0.05 and 0.1 (marginal effects) are italicized.

#### Trial duration

Averaged over all doses and sessions biperiden did not differentially affect trial duration in the two groups (Doses: F_4,52_ = 0.43, p = 0.7834; Pig breed X Doses interaction: F_4,52_ = 1.00, p = 0.4143; Pig breed X Doses X Sessions interaction: F_4,52_ = 0.88, p = 0.4842). Trial duration tended to increase with increasing biperiden dose (Sessions: F_1,13_ = 0.35, p = 0.5646; Dose X Sessions interaction: F_4,52_ = 2.23, p = 0.0784; Pig breed lines X Sessions interaction: F_1,13_ = 1.17, p = 0.2997). Analysis of data for the minipigs revealed that the higher doses of biperiden increased trial duration more than did the lower doses (Doses X Sessions interaction: F_5,35_ = 3.34, p = 0.0143; Doses: F_5,35_ = 0.78, p = 0.5712; Sessions: F_1,7_ = 0.00, p = 0.9525). Trial duration is shown in Figure 3C.

#### Latency to first rewarded hole visit

As is shown in Figure 3D, the minipigs took longer to find the first food reward than did the conventional pigs (F_1,13_ = 8.05, p = 0.0140), but biperiden did not affect the time it took in either group (Dose: F_4,52_ = 1.08, p = 0.3772; Pig breed X Dose interaction: F_4,52_ = 1.42, p = 0.2416). However, across all doses tested, the pigs took longer to find the first food reward when they were treated with biperiden than in the preceding control session (Session: F_1,13_ = 14.55, p = 0.0021), with a marginal Dose X Sessions interaction suggesting that the higher, rather than lower, doses of biperiden tended to increase the time it took pigs to find the first food reward (F_4,52_ = 2.54; p = 0.0508). This effect was similar for the two groups of pigs (Pig breed X Sessions interaction: F_1,13_ = 0.01, p = 0.9236; Pig breed X Dose X Sessions interaction: F_4,52_ = 0.04, p = 0.9968). Analysis of the data for the minipigs confirmed that this effect of biperiden on the time taken to find the food reward was not dose related (Dose X Sessions interaction: F_5,35_ = 1.16, p = 0.3476).

## Discussion

Both the conventional pigs and the Göttingen minipigs learned the holeboard task. This finding corroborates and extends earlier studies by Arts et al. [8] and Gieling et al. [9] and confirms our hypothesis. After intensive training (about 100 trials), the pigs reached nearly errorless asymptotic WM and RM performance. Moreover, all pigs had a higher level of performance than we have ever seen in rats, even after more that 400 training trials [18]. The holeboard performance and motivation of minipigs were not different from those of conventional pigs, showing that both types of pig can be used in cognitive research. This is in marked contrast to the conclusion drawn by Downes [30] based on a study by Manton [14], that “the results of testing for learning and memory in minipigs were equivocal and ultimately disappointing”. We found that minipigs could acquire the spatial holeboard discrimination task, suggesting that Downes’ conclusions concerning testing the learning and memory capacity of minipigs, using the holeboard task are premature.

The cholinergic system is involved in spatial discrimination learning [15], and holeboard-type tasks are sensitive to manipulation of cholinergic neurotransmission with so-called cognition enhancers or cognition impairers (for a recent review see [10]). Biperiden, an M1 receptor antagonist, is suggested to act as a cognition impairer [7,17,19], but we found only marginal effects on RM and WM at very high oral doses (5 to 20 mg.kg^-1^). The WM performance of the conventional pigs appeared to be unaffected by biperiden, whereas the drug appeared to differentially affect WM performance in the minipigs, with the lowest dose of biperiden marginally improving WM performance and the highest dose marginally impairing WM performance (see Table 1). On the basis of the effects of the cognition impairers scopolamine and MK-108 in well-trained rats [18], we expected that biperiden would transiently affect WM and RM performance. The lowest dose of biperiden that impaired RM in both groups of pigs was 5 mg.kg^-1^, which is in the dose range that was found to affect cognition in rats [19]. Thus although we had expected that biperiden would transiently impair spatial memory in conventional pigs and age-matched minipigs, once they have learned the cognitive holeboard task, we cannot unambiguously conclude that this is the case. We can conclude that the effects found on RM are comparable for both breeds, although the highest dose could not be tested in conventional pigs.

It may be difficult to impair memory performance once a task has been learned to almost perfection, but previous studies with the conefield task, a variant of the holeboard task, have shown that the near-maximal, asymptotic WM and RM performance of rats could be decreased with the cognition impairers scopolamine and MK-801. In the drug-free session following each of the scopolamine or MK-801 sessions, the WM and RM performance returned to control level [18]. Although the rats reached an asymptotic performance, they did not reach the maximum performance possible (ceiling level). In the current study, the peak performance of the pigs was close to the maximum performance level possible. In order to detect cognition-impairing effects, it might be appropriate to give biperiden earlier, before pigs reach an asymptotic level of performance, or the task could be made more difficult (e.g. hide 5 instead of 4 rewards). This is supported by the observation that while the performance of laboratory animals in the conefield task can be altered by cognition impairers [18], performance in other spatial learning and memory tasks, such as the Morris water escape task, is unresponsive to the effects of cognition impairers once the task has been learned to an asymptotic level [31]. In a study involving a well-learned operant task and rats [32], biperiden at doses of 0.25 and 0.5 mg.kg^-1^ increased the number of nonreinforced responses and decreased the number of reinforcements obtained. Doses exceeding 0.5 mg.kg^-1^ already led to long pauses in responding and omissions to respond. In pigs, doses equal to and exceeding 5 mg.kg^-1^ tended to increase trial duration and the time taken to find the first food reward. The longer trial duration may have been caused by a longer time taken to visit the first hole and an increase of the number of erroneous hole visits before all the baits were found (RM errors).

Our findings suggest that conventional and minipigs differ in their sensitivity to the disruptive effects of biperiden with only the conventional pigs starting to refuse the higher doses. However, this apparent difference might have been caused by differences in body composition. Leanness significantly affects the volume of distribution of a drug and hence its adverse responses. Moreover, biperiden is highly lipophilic, rapidly entering the brain, but slowly entering fat tissue, from where it is slowly cleared [33]. Although we did not measure the proportion of body fat, we presumed that the conventional pigs were leaner than the minipigs. If this is the case, then minipigs are not necessarily less sensitive to the pharmacological effects of the biperiden, but instead have different pharmacokinetics. Although we found that biperiden did not impair retrieval of well-consolidated information from RM, and within-trial WM, it remains to be seen whether biperiden affects the learning process and/or memory consolidation. This can be assessed by administering biperiden before or immediately after the daily training trials during acquisition of the holeboard task. Biperiden has been found to impair consolidation and retrieval in a passive avoidance task with rats [26,34].

Our data confirm that biperiden is safe, even after administration of very high doses. The pigs were treated orally with up to 20 mg.kg^-1^: biperiden, a dose approximately 400-times higher than the therapeutic dose usually used in humans. We observed only mild non-cognitive adverse effects at the highest doses tested, such as mild signs of dry mouth [29], a typical side effect of anticholinergic drugs [16]. Liquid reinforcements could be used to get around the dry-mouth problem. It remains to be determined whether the reduced RM performance observed with the highest dose of biperiden really reflects cognitive deficits caused by a central action of biperiden or noncognitive adverse drug reactions. In the latter case, biperiden would not fulfill the requirements of a pharmacologically active cognition impairer in pigs [31]. A potential limitation of the study is that none of the conventional pigs ate the mixture containing the highest dose of biperiden and one did not did not eat the mixture containing 15 mg.kg^-1^ biperiden. This reluctance to eat is unlikely to have been due to a dry mouth, because this side effect developed about 30 minutes after drug ingestion. Dry mouth might have diminished the attractiveness or palatability of the M&M rewards. However, all Göttingen minipigs ate the food containing biperiden, even though they showed symptoms of dry mouth. The large volume of the mixture of crushed tablets, pig feed, and honey (the latter needed to stimulate pigs to eat the tablet mixture) may have delayed the absorption of biperiden, such that peak plasma levels, which were expected approximately 1–1.5 hours after drug administration on the basis of published pharmacokinetic data [20], were reached later. This would have led to underestimation of the ability of biperiden to affect spatial memory. In the future, it might be more effective to administer pure biperiden, so that less “filler” is needed.

The present study did not provide evidence to support the conclusion drawn by Klinkenberg and Blokland [19] that M1 receptor antagonists can be considered an alternative to scopolamine as cognition impairer, at least if conventional or minipigs are used as subjects. We suggest that biperiden should be evaluated further as putative cognition impairer in pigs, but using a different way to administer the drug. On the basis of our findings, a counterbalanced design could be used in a future study, e.g. involving the doses 1, 3, and 10 mg.kg^-1^. The learning task could be made more difficult, and RM performance should be monitored daily, so that treatment can be started before performance reaches an asymptotic level, to prevent a ceiling effect. On the basis of our findings, minipigs can be used instead of conventional pigs or can serve as a translational model for other species. The effectiveness of biperiden treatment in conventional and minipigs clearly needs to be evaluated further before definitive conclusions can be drawn about the ability of biperiden to impair cognition in this species.

## Competing interests

Financial support for programming the software for the holeboard apparatus and in kind (minipigs) were provided by *Ellegaard Göttingen minipigs* (Denmark). N.C. Ganderup is employee of Ellegaard. All other authors declare no conflict of interest. The setup of the experiment was not designed neither influenced by *Ellegaard Göttingen minipigs*.

## Authors’ contributions

ETG has made substantial contributions to conception and design, acquisition of data, analysis and interpretation of data and has been involved in drafting the manuscript. WW has made substantial contributions to acquisition and analysis of data. RW has made substantial contributions to acquisition and analysis of data. REN has been involved in drafting the manuscript and did language corrections. NCG has been involved in drafting the manuscript and data interpretation. FJS has made substantial contributions to conception and design, analysis and interpretation of data and has been involved in drafting the manuscript. All authors read and approved the final manuscript.
